# Folic acid fortification of double fortified salt

**DOI:** 10.1038/s41598-021-93194-9

**Published:** 2021-07-15

**Authors:** Oluwasegun Modupe, Juveria Siddiqui, Akhila Jonnalagadda, Levente L. Diosady

**Affiliations:** 1 200, College Street, Toronto, ON M5S 3E5 Canada; 2 Department of Chemical Engineering and Applied Chemistry, University of Toronto, Toronto, ON Canada

**Keywords:** Chemical engineering, Process chemistry, Reaction kinetics and dynamics, Food nanotechnology, Nutrition

## Abstract

The addition of folic acid to Double Fortified Salt (with iron and iodine) aims to simultaneously ameliorate three major micronutrient deficiencies in vulnerable populations. To make Triple Fortified Salt, we added folic acid to the iodine solution (first method) and the iron premix (second method) that are used to fortify salt with iron and iodine. When added through the solution, sodium carbonate was needed to dissolve folic acid and to adjust pH. Alternately, folic acid was added either to the iron core or sandwiched between the core and TiO_2_ layer of the iron premix. Folic acid and iodine were stable in all cases, retaining more than 70% of the added micronutrients after six months at 45 °C/60–70% relative hu. Adding folic acid to the premix's iron core is preferred as folic acid retention was slightly higher, and the added folic acid did not impact the salt's colour. The additional cost for adding the micronutrients to salt is about 27¢/person per year. Folic acid in the fortified salt made with the preferred method was stable in cooking and did not affect selected cooked foods' sensory properties. The technology is a cost-effective approach for simultaneously combating iron, iodine, and folic acid deficiencies.

## Introduction

Vitamin B_9_ (folate) plays a vital role in the metabolism of nucleic acids and proteins^1,2^. Its deficiency is a major cause of neural tube defects^3^. In some cases, the deficiency of vitamin B_9_ also can cause cancer, cardiovascular diseases, and impaired cognitive function. Folic acid is the synthetic form of naturally existing folate. It only differs from folate in that it has only one glutamic acid moiety. This difference makes folic acid more stable and readily bioavailable^4^.

The increasing prevalence of neural tube defects, a direct consequence of vitamin B_9_ deficiency in women of reproductive age, led many developed countries, including Canada and the USA, to mandate the fortification of flour with folic acid^5,6^. This action has resulted in a drastic reduction in the prevalence of neural tube defects in those countries^7^. Castillo-Lancellotti et al.^8^, Atta et al.^9^, and Centeno-Tablante et al.^10^, in their extensive review studies, showed a significant reduction in the spina bifida prevalence in countries with folic acid fortification program. Unfortunately, large rural populations in developing countries do not have access to or cannot afford these fortified foods. Therefore adding folic acid to table salt that is ubiquitously consumed has an immense potential public health benefit.

The addition of folic acid to the Double Fortified Salt (salt fortified with iron and iodine) will be more economical and practicable than having a separate folic acid fortified salt and allow simultaneous amelioration of iron, iodine, and folic acid deficiencies that can prospectively impact reproductive aged-women and children's health and or growth. The existing technology for adding micronutrients to salt involves adding some micronutrients as a premix, while others are sprayed as a solution on salt^11–14^. With the technology developed at the University of Toronto for the double fortification of salt with iron and iodine, ferrous fumarate, as the iron source, was added to salt as an extruded and microencapsulated particle that matches the size and colour of salt; a solution of potassium iodate, the iodine source, was sprayed unto the salt^15^. This technology provides two potential paths for which folic acid can be added to salt—either through the extruded iron microcapsule or the iodine spray solution.

Folic acid can impart a bright yellow colour to salt if added directly with the iodine solution^16^. Additionally, since folic acid is only sparingly soluble in water, a solvent system is thus required to dissolve a high concentration of folic acid. Folic acid is susceptible to oxidative degradation^16,17^. While potassium iodate has oxidative potential, ferrous fumarate has reductive potential. It was crucial to investigate how these constituents of Double Fortified Salt would affect folic acid stability and, ultimately, establish how folic acid is best added to salt.

McGee et al.^18^ described a process for adding iodine and folic acid to salt, where 1–3 g folic acid and iodine were dissolved in a 100 mL bicarbonate buffer solution adjusted to pH 9. The solution was sprayed on the salt so that the concentrations of iodine and folic acid in the salt were 50 ppm each. Modupe et al.^16^ also described the addition of iron, iodine, and folic acid to salt. While iodine and folic acid were added as a solution sprayed unto salt, iron was added as an extruded and encapsulated ferrous fumarate. A buffer solution was not used,instead, 1% folic acid and 2% iodine were dissolved in 0.1 M sodium carbonate solution. The solution (5 mL) was sprayed on 2 kg salt mixed with a ribbon blender. The salt was air-dried and mixed with the microencapsulated ferrous fumarate (10 g).

The present study was carried out to evaluate the methods of adding folic acid to Double Fortified Salt. The study's objective was to develop Triple Fortified Salt (TFS) that retains at least 70% of the added micronutrients for at least six months, without significantly impacting salt or cooked foods' sensory properties.

## Materials and methods

### Materials

Ferrous fumarate (food grade) was obtained from *Dr. Paul Lohmann Chemicals (Emmerthal, Germany)*; soy stearin (SS) was obtained from *JVS Food Pvt, (India);* hydroxypropyl methylcellulose (HPMC) was obtained from *Dow Chemical Company (Midland, Michigan USA)*; titanium (IV) oxide was obtained from *ACROS Organics (Fair Lawn, New Jersey, USA)*; folic acid was obtained from *Bulk Pharmaceuticals Inc., (Toronto, Ontario, Canada);* potassium iodate was obtained from *Sigma–Aldrich Chem (Oakville, Ontario, Canada);* absolute ethanol and dichloromethane were obtained from *Thermo Fischer Scientific (Mississauga, Ontario, Canada);* sodium carbonate, potassium iodide and starch indicator were obtained from *Caledon Laboratory Ltd (Georgetown, Ontario, Canada);* sulfuric acid was obtained from *EMD Chemicals Inc. (Oakville, Ontario, Canada);* and 1.0% sodium thiosulfate solution was obtained from *VWR International, (Mississauga, Ontario, Canada); v*egetable shortening, semolina, rice *grains* and ingredients used for cooking were obtained from *Walmart (Toronto, Ontario, Canada).*

All chemicals used for the fortification of salt were food-grade, while those used for analysis were ACS grade.

### Formulation of spray solutions

#### Iodine solution

A potassium iodate solution (3.37% ^w^/_v_), containing 2% ^w^/_v_ iodine, was prepared in a 100 mL volumetric flask with RO water.

#### Folic acid and iodine solution

Solutions of folic acid and iodine (1–3% ^w^/_v_) were prepared with sodium carbonate buffers (0.1–0.3 M); the impact of sodium carbonate buffer concentration on folic acid solubility was investigated. The second set of three solutions contained 1% folic acid + 1% iodine, 2% folic acid + 2% iodine and 1.8% folic acid + 3% iodine. The solutions were formulated with 0.2 M sodium carbonate buffer, and the stability of iodine and folic acid was monitored for 2 months. The third set of solutions containing 0.5–1% ^w^/_v,_ folic acid, and 2% ^w^/_v_ iodine was made using sodium carbonate solution (0.1 M) instead of the carbonate buffer. The sodium carbonate solution was used to adjusted the pH of the solution from 7 to 10. The pH of the solutions was measured with a VWR Scientific Model 8000 pH meter.

The spray solutions were tightly secured in scintillation vials and were stored in temperature-controlled incubators (25, 35, 45 °C). The stability of iodine and folic acid in the spray solutions were monitored for two months. The stability of iodine and folic acid in the solution was expressed as a percentage of the micronutrients in the freshly prepared solution. Given the precipitation of folic acid from some higher folic acid concentration solutions, the 2% iodine and 1% folic acid solution adjusted to pH 9 with sodium carbonate solution was used for salt formulation.

### Formulation of micronutrient premix

The iron premix was formulated as described by Li et al.^19,20^ and Modupe et al.^16^. Ferrous fumarate (800 g) was thoroughly mixed with semolina (200 g), vegetable shortening (25 g), and water with a Kitchen Aid Mixer. The dough was preconditioned for 2–3 h. The dough was extruded with a La Monferrina P12 pasta extruder. The extrudate was cut, and size screened to match the size of salt (300–600 µm). The extrudate was colour masked by dusting titanium dioxide (15% ^w^/_w_) on the surface of the extrudate and finally coated with 5% (^w^/_w_) HPMC and 5% (^w^/_w_) soy stearin, respectively.

Two models were designed for the iron-folic acid premix: (a) the coextrusion of iron and folic acid such that both iron and folic acid were in the core of the premix, and (b) the sandwiching of folic acid between titanium dioxide layers such that the iron core was separated from the folic acid. For iron and folic acid coextrusion, the same process described for making iron premix was followed. For this purpose, 7 g of folic acid was added and mixed with the dough before preconditioning and extrusion. In the second design, the process of making iron premix was followed until the colour masking step. There were two colour masking steps before the coating steps—the first colour masking step created a thin layer between iron extrudate and the folic acid layer, and the second colour masking step masked the yellow colour of folic acid. After the first colour masking step, either a homogenous suspension of folic acid (0.52% ^w^/_v_) in a 2.5% ^w^/_v_ HPMC (dissolved in a 1:1 absolute ethanol: dichloromethane solvent) was sprayed on the TiO_2_ masked iron extrudate tumbling in a pan coater or a suspension of folic acid in water was mixed thoroughly with colour masked iron extrudate. The iron-folic acid particles obtained from the two approaches were again colour-masked and coated with HPMC and soy stearin, as described earlier.

### Formulation of fortified salt

Non-iodized refined salt (about 400 µm diameter), obtained from *Sifto Canada Corp, was mixed in a ribbon blender to break any salt lumps*. A micronutrient(s) solution, either of iodine or iodine and folic acid (2.5 mL/ kg salt), was sprayed onto the salt inside the ribbon blender and mixed. This corresponds to 50 ppm iodine and or 12.5–25 ppm folic acid in the salt. The mixing operation was stopped intermittently to remove any solution stuck to the blade of the blender. The salt was mixed with the solution for about 20 min. The salt was then collected and spread on aluminum foil for air drying.

The air-dried salt was mixed with iron or iron-folic acid premix. For a 2 kg batch, 10 g of the premix was mixed with 1990 g of salt in the ribbon blender. The mixing ensured uniform dispersion of 0.5% ^w^/_w_ premix in the salt. The iron premix concentration in the iron premix was about 20% ^w^/_w,_ such that the formulated salt contained 1000 ppm iron, 50 ppm iodine, and 12.5–25 ppm folic acid.

### Handling and storage of fortified salt

The salt samples were divided first into four portions by a glass divider; the fourth portion was shared over the other three portions. The glass divider ensured a randomized division of the salt into the three portions. They were packed in Zip-LockTM polyethylene freezer bags and stored at incubators with controlled temperature and humidity (25, 35, and 45 °C; 60–70% RH (relative humidity)). Also, the incubator was in complete darkness so that the impact of light was minimized.

### Stability study of fortified salt

The stability of folic acid and iodine in some of the premix samples and each salt sample were evaluated at 0, 0.5, 1, 2, 4, 6, and 12 months. The amount of iodine and folic acid in the salt was reported as the percentage of the micronutrients in the freshly prepared samples. Iodine and folic acid in the salt were quantified as described by Modupe et al.^16^ and Modupe et al.^21^, respectively.

### Evaluation of the colour of the premix

The colour of iron-folic acid premix was used to judge the homogenous distribution of folic acid in the premix. The colour was observed from the image obtained with an iPhone 8 camera.

### Elucidation of the chemistry folic acid in spray solution

The mass spectrum of folic acid in sodium carbonate solution was obtained from Thermo Q-Exactive Mass Spectrometer. The mass spectrum was obtained for folic acid in solutions that contained just folic acid or folic acid and potassium iodate.

### Kinetic study of the degradation of folic acid and iodine in triple fortified salt

For some salt samples, the amounts of micronutrients were determined at 0.5, 1, 2, 4, and 6 months of storage. The data obtained from these studies were used to extrapolate kinetic data of the degradation of micronutrients in the salt over the period. The expression of zero (Eq. 1) and first-order kinetics (Eq. 2) were used. The best fit was selected. The kinetic data obtained were validated with data obtained from a 12-month stability study. The same data was used to extrapolate the equation to predict micronutrients' long-term stability in the salt.

1 $$\left[ A \right]_{t} = - kt~ + ~\left[ A \right]_{0}$$

2 $$ln\left[ A \right]_{t} = ~ - kt~ + ln\left[ A \right]_{0}$$

#### Effect of cooking on the stability of folic acid

The degradation of folic acid during cooking was evaluated. Boiled rice and Bondi raita (a yogurt-based Indian dish) were selected. A known amount (~ 4–5 g per kg food) of unfortified and fortified salt was used. The rice was boiled for 20 min while the fermentation of Bondi raita was allowed to continue for 30 min. Folic acid was extracted from the food and quantified by HPLC, as described in Table 1.

**Table 1:** HPLC Conditioned for Folic Acid and Vitamin B_12_ Analysis.

Column	Kinetex, 2.6 µm, C18, 100 Å, LC column 100 × 4.6 mm			
Dimensions	100 × 4.6 mm ID			
Elution type	Gradient			
	Acetonitrile			
Elution B:	20 mM phosphate buffer pH 3			
Gradient profile	Step No	Time (min)	Sol. A	Sol. B
	0	0.5	5	95
	1	5.0	25	75
	2	5.00	5	95
	3	5.00	75	25
	4	5.00	5	95
Flow rate	1 mL/min			
Col. Temp	Ambient			
Detection	UV–Vis Abs.-variable wave.(UV) @ 278 & 360 nm (22 °C)			
Run time	20 min			
Injection volume	20µL			

## Results and discussion

In the process described by Li et al.^15^ for the double fortification of salt, iodine as a solution of potassium iodate was sprayed onto the salt, and iron was added as extruded and microencapsulated ferrous fumarate. The addition of folic acid through the iodine solution was thought to be the easiest path for adding folic acid to salt. Hence, in a first step to making salt fortified with folic acid, a solution of folic acid and iodine was added to salt^22^. However, the salt's high moisture content (2.9%) due to the low concentration of folic acid and iodine (0.35%, each) in the spray solution accelerated iodine loss in the salt. Therefore, a higher concentration of folic acid and iodine (at least 1%, each) was formulated. With this spray solution, less solution was added to salt, resulting in low moisture content (0.06% or less).

### Preliminary formulation of spray solution

**Figure 1:**
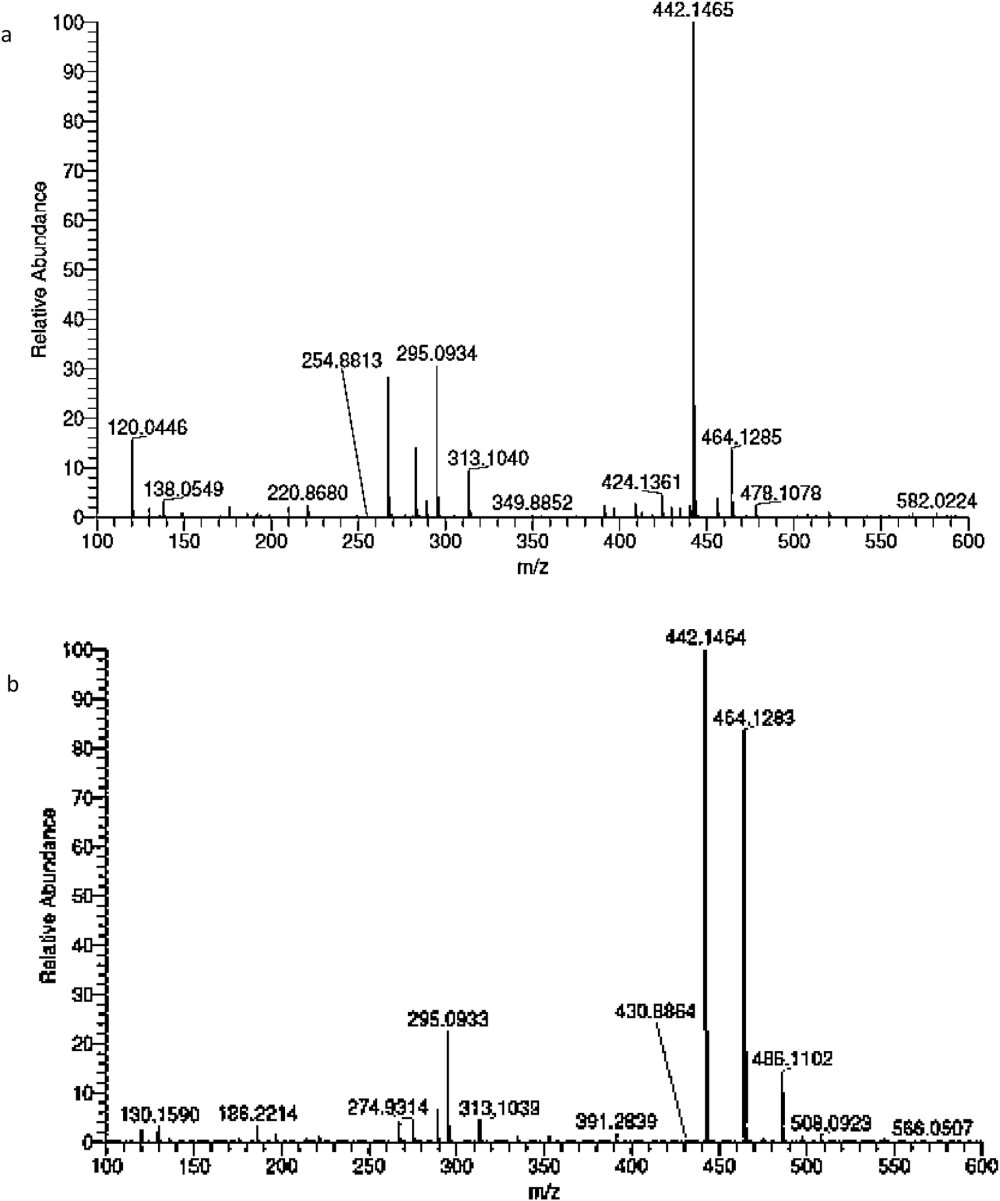
Mass spectrum of folic acid in spray solution (**a**) folic acid in sodium carbonate solution (**b**) folic acid and potassium iodate in sodium carbonate solution.

The impact of sodium carbonate buffer concentration on folic acid solubility in the spray solution was evaluated. The solubility of folic acid in the solution increased as the carbonate buffer concentration increased; at least 0.2 M sodium carbonate buffer was required to dissolve 3% folic acid. The MS spectra of folic acid in the solution showed the formation of mono- and disodium salts of folic acid. The relative abundance of the disodium salt was higher in the solution that contained potassium iodate (Fig. 1). The formation of sodium salt was responsible for folic acid solubility and explained why a higher concentration of sodium carbonate buffer is required to dissolve 3% folic acid.

The stability studies carried out on the second set of solutions (that contained 1% folic acid + 1% iodine, 2% folic acid + 2% iodine, and 1.8% folic acid + 3% iodine) showed that both iodine and folic acid were very stable in the solution. Less than 20% of the added micronutrients were lost after 2-month storage, even at 45 °C. This result was consistent with those reported by McGee et al.^18,22^. While increasing the iodine concentration resulted in a significant increase in iodine and folic acid stability, increasing folic acid concentration did not impact iodine and folic acid stability in any particular pattern. The relatively higher folic acid stability observed in the solution that contained higher iodine (3%) may be due to the formation of more disodium salt of folic acid. At the point of preparation, 0.2 M sodium carbonate buffer dissolved folic acid, irrespective of the amount of folic acid added (1, 1.8, or 2% folic acid). However, in the solutions that contained more than 1% ^w^/_v_ folic acid, some of the folic acids precipitated out from the solution, which can be a significant problem for small salt plants that store their spray solutions for up to a month.

### Stability of iodine and folic acid in the optimized spray solution

To prevent folic acid precipitation, the spray solution was reformulated, reducing the folic acid concentration to 0.5–1%, and the buffer solution used in the previous method was replaced with a sodium carbonate solution (0.1 M). The solution of sodium carbonate itself is a buffer as it dissociates to sodium and bicarbonate ions. The iodine concentration was maintained at 2% in the spray solution to be consistent with industrial practice. The change from the buffer (prepared with sodium carbonate and sodium bicarbonate) reduced the number of steps required for making the spray solution. In all the samples, 70–100% of folic acid and iodine were retained after two months of storage (Table 2).

**Table 2:** Stability of folic acid and iodine in the optimized spray solution.

Types of solution	Folic acid			Iodine		
	25 °C	35 °C	45 °C	25 °C	35 °C	45 °C
0.5% FA	82.4 ± 3.4	82.0 ± 1.9	68.8 ± 6.7			
1% FA	92.1 ± 2.3	84.4 ± 6.9	71.5 ± 3.4			
0.5% FA + 2% I	91.1 ± 2.1	88.4 ± 4.9	74.4 ± 2.2	94.7 ± 4.7	92.7 ± 0.6	89.4 ± 4.6
1% FA + 2% I	88.7 ± 4.7	89.3 ± 2.6	80.2 ± 5.2	96.5 ± 4.0	94.4 ± 1.0	88.6 ± 2.7
2% I				100.0 ± 1.0	96.5 ± 5.1	93.1 ± 1.2

FA = folic acid, I = iodine; the pH of the solutions were adjusted to pH 9.

The concentration of folic acid in the spray solution did not significantly affect folic acid and iodine stability in the spray solutions, except at 25 °C (Table 2). At this temperature, the percentage of folic acid retained in the spray solution, containing only 0.5% folic acid, was significantly lower than other solutions. This trend was also was reported by McGee et al.^18^. As shown earlier, potassium iodate accelerated the formation of sodium salts of folic acid in the solution. This may be responsible for the improved stability of folic acid in solutions that contained potassium iodate.

pH had a significant effect on the solubility of folic acid in the KIO_3_ solutions. After a few weeks, folic acid precipitated out of the solutions at pH 7 and 8. This observation was consistent with the study of Taub and Lieberman^23^, who found that folic acid solution at pH 6 turned cloudy after a few days. Folic acid in solutions adjusted to pH 9 and 10 did not precipitate even after a few months. Hence, the pH of subsequent solutions was maintained at ≥ pH 9. In order to obtain pH 9, 0.742 g sodium carbonate, 3.37 g potassium iodate, and 1 g folic acid were dissolved in 100 mL of water.

### Stability of iodine and folic acid in triple fortified salt (TFS) after 6-months storage

The first attempt on triple fortification of salt was reported by McGee^22^, who formulated TFS by spraying 0.35% iodine and folic acid on salt and adding iron premix prepared by a one-step agglomeration and encapsulation using spray drying^24^. The idea was that the relatively smaller iron premix particles would adhere to the salt particles' surface. The lower concentration of folic acid and iodine in the spray solution led to adding more liquid to the salt (30 mL/kg); hence, increasing the salt's moisture content. The high moisture content of the salt accelerated the rate of loss of iodine. The iron premix colour was not acceptable as it formed grey spots in the salt^24^.

In contrast to the technology developed by McGee^22^, a higher concentration of folic acid and iodine (05–1% and 2%, respectively) was used. The spray solution volume was drastically reduced from 30 to 2.5 mL/kg salt. The newly fortified salt's moisture content was 0.06% compared to 2.9% of the fortified salt formulated by McGee^22^. The premix, prepared by forming extrusion and microencapsulation similar to that developed by^19,20^, was used in place of the premix formulated by spray drying. The similar particle size and density of the premix and salt were produced ensured that the iron premix would not segregate from the salt.

Table salt stays in the distribution channels for an average of 2 months^25^. Also, the target population buys a small amount of salt, typically consumed within two months. Hence, the goal is to have at least 70% of the micronutrients retained in the fortified salt after 6-month storage. This was achieved; after 6-month storage, 70–85% of folic acid and 85–95% of iodine were retained in all the samples, even at 45 °C and 60–70% RH (Table 3). This result confirmed that the technology could deliver iron, iodine, and folic acid simultaneously through salt. Given the traditional distribution channels of salt, Triple Fortified Salt has the potential of reaching millions of vulnerable households that otherwise may not have access to diets with sufficient iron, iodine, and folic acid.

**Table 3:** Stability of folic acid and iodine in T after 6-month storage.

Types of		Retention of folic acid (%)			Retention of iodine (%)		
Sol	Prem	25 °C	35 °C	45 °C	25 °C	35 °C	45 °C
I	H&S	–	–	–	93.2 ± 2.9	90.3 ± 3.9	92.3 ± 6.8
C	–	82.3 ± 6.1	80.4 ± 5.9	78.9 ± 3.5	95.3 ± 3.4	92.9 ± 3.6	89.2 ± 6.2
D	–	81.1 ± 1.2	77.3 ± 0.8	72.9 ± 3.3	92.2 ± 1.5	89.8 ± 2.1	86.8 ± 1.4
C	H&S	83.5 ± 0.5	82.2 ± 2.9	83.5 ± 0.5	89.6 ± 3.2	89.7 ± 3.9	87.4 ± 1.8
D	H&S	80.6 ± 1.5	77.8 ± 1.2	73.2 ± 1.5	93.1 ± 1.5	94.0 ± 4.3	93.0 ± 1.4

Sol. = solution: C = 0.5% folic acid + 2% iodine, D = 1% folic acid + 2% iodine, and I = 2% iodine. Prem. = iron premix: H&S = iron premix coated with 5% HPMC and 5% soy stearin.

The direct addition of folic acid on the surface of the salt resulted in bright yellow salt. Although the reduction in folic acid concentration in the salt (12.5 ppm) significantly reduced the salt's yellow colour, it was still visibly yellow. The colour may reduce the end-users' acceptance of the salt since colour is an essential factor for selecting food products by consumers^26^. Hence, technology to eliminate this colour change was investigated.

### Optimizing the process of triple fortification of salt

**Figure 2:**
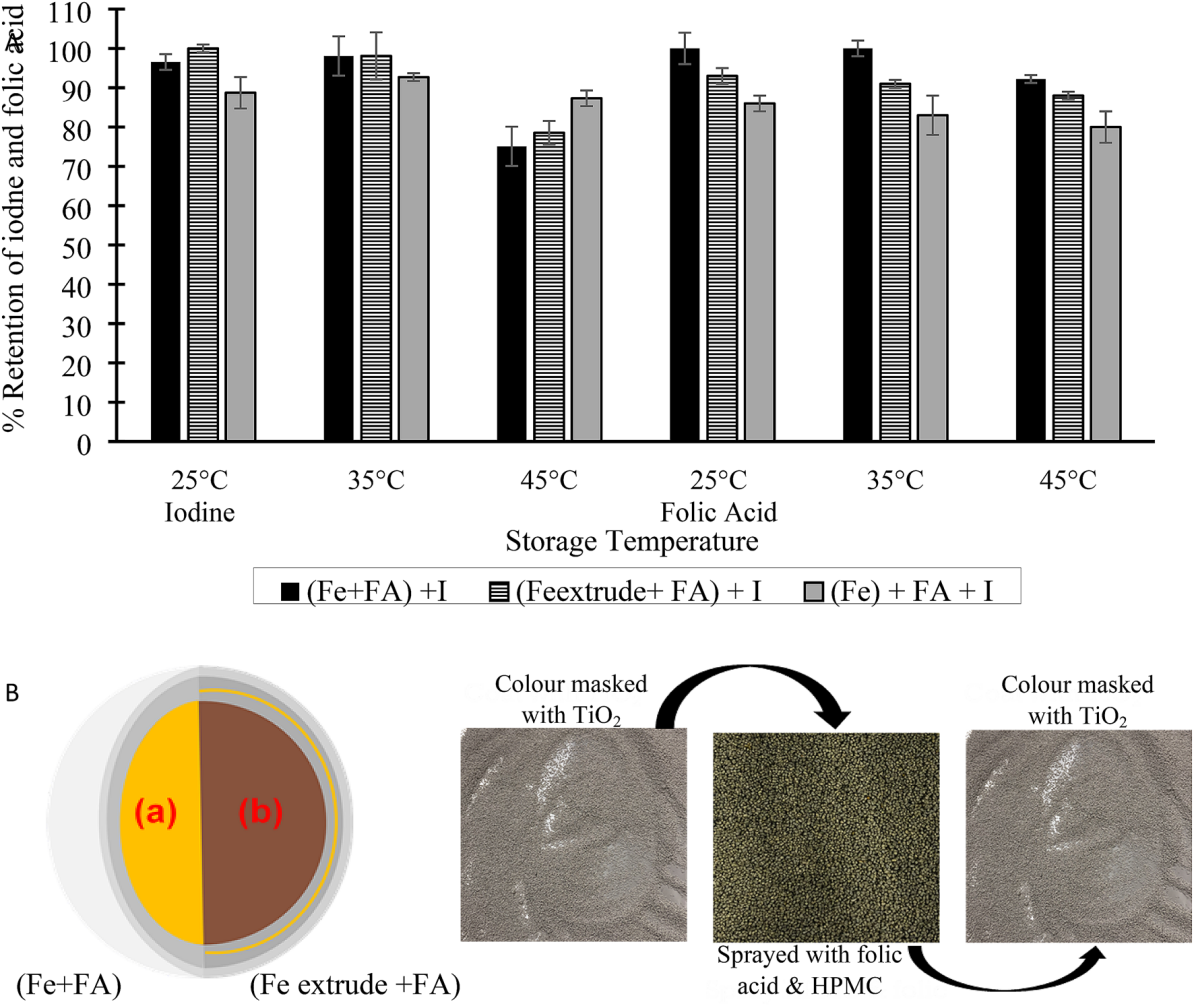
(**A**) Stability of iodine and folic acid in TFS formulated with Fe-FA premix. (**B**) Schematic of the two designs for iron-folic acid premix and how one of the premix samples (Fe extrude + FA) was made (Fe + FA) has both iron and folic acid in the core of the premix; (Fe_extrude_ + FA) has folic acid separated by a tiny layer of TiO_2_ in the premix; (Fe) has only iron in the premix, and folic acid and iodine was added as a solution.

Folic acid was added to the iron premix to form a Fe-FA premix. There were two designs for Fe-FA premix—either iron and folic acid were added to the core of the premix (Fe + FA) or folic acid was separated from the iron in the core by a thin layer of TiO_2_ (Fe_extrudate_ + FA), as illustrated in Fig. 2B. Iron and folic were coextruded to have both micronutrients in the core of the premix. Coextruding iron and folic acid was a straight forward process; it only involved adding folic acid to ferrous fumarate before extrusion. Separating folic acid from the core was achieved by two different routes:

- adding folic acid as a uniform suspension in water to colour masked iron extrudate, or
- spraying a folic acid suspension in 2.5% HPMC (in a 1:1 ethanol and dichloromethane solvent system) on color-masked iron extrudate tumbled in a pan coater.

The solvents' ratio that made up the solvent system is vital to having a uniform suspension of folic acid that is volatile enough for pan coating. A 1:1 dichloromethane: ethanol solvent system was used. More dichloromethane caused folic acid to settle at the bottom of the spray flask, while more ethanol caused the colour masked iron extrudate to clump inside the pan coater. Spraying folic acid suspension in 2.5% HPMC (Route b) resulted in a more uniform distribution of folic acid than the other method (Route a).

The iron-folic acid premix (Fe_extrude_ + FA) with uniform distribution of folic acid (made by Route b) and the coextruded iron and folic acid premix (Fe-FA) were subsequently used to formulate TFS. In the optimized process, folic acid will no longer impact the TFS color, as folic acid was hidden with iron by the colour masking and coating agents of the premix. Over 75% of the added folic acid and iodine were retained after 6-month storage. The loss of iodine in the salt did not follow any particular trend (Fig. 2A). Folic acid was more stable in the (Fe + FA) premix than the (Fe_extrudate_ + FA) premix. TiO_2_ being in contact with folic acid may have initiated photocatalytic degradation, which led to the significant loss of folic acid. Putting folic acid in the opaque core of the ferrous fumarate (Fe + FA) prevented photocatalytic degradation. Folic acid was more stable in the TFS formulated with Fe-FA premix than in TFS formulated by spraying folic acid and iodine solution on salt. Iron seems to have enhanced the stability of folic acid in the salt. The same pattern was shown by^19,20^ in fortified rice that contained folic acid and iron. McGee et al.^18^ suggested that folic acid loss in a salt fortified with iodine and folic acid is due to oxidative stress. The stability of folic acid in the TFS confirms the oxidative degradative pathway of folic acid in salt postulated by Modupe et al.^16^ and Modupe^27^. The reductive potential of ferrous iron may have prevented the oxidative degradation of folic acid in the salt. Aside from the enhanced stability of folic acid in the (Fe + FA) premix, it is easier to make than the (Fe_extrudate_ + FA) premix. Going forward, TFS should be formulated by adding folic acid and the iron source (usually ferrous fumarate) as microencapsulated coextrudate, and iodine added by the traditional method of spraying potassium iodate solution. The TFS will deliver 200% iodine's RDA, 56% iron RDA and 100% folic acid RDA based on the consumption of 10 g of salt per day^28^.

Other microencapsulation technologies could have been considered for folic acid in this study, for instance, the electrohydrodynamic technology, described by Bakhshi et al.^29^, and the spray drying technology described by Assadpour et al.^30^. These techniques cannot produce the microcapsule's desired size, and their introduction into the fortification process will increase the plant's capital cost as none of these technologies are used in salt fortification. The technology developed can be used in the existing setup of plants used for making Double Fortified Salt. Also, it is uncertain whether the technology described by Bakhshi et al.^29^ or Assadpour et al.^30^ can effectively mask the colour of ferrous fumarate or folic acid. Romita et al.^24^ clearly showed that spray drying technology could not effectively mask the ferrous fumarate's dark brown color.

### Calculation and validation of the kinetic parameters for the stability of iodine and folic acid in TFS

**Figure 3:**
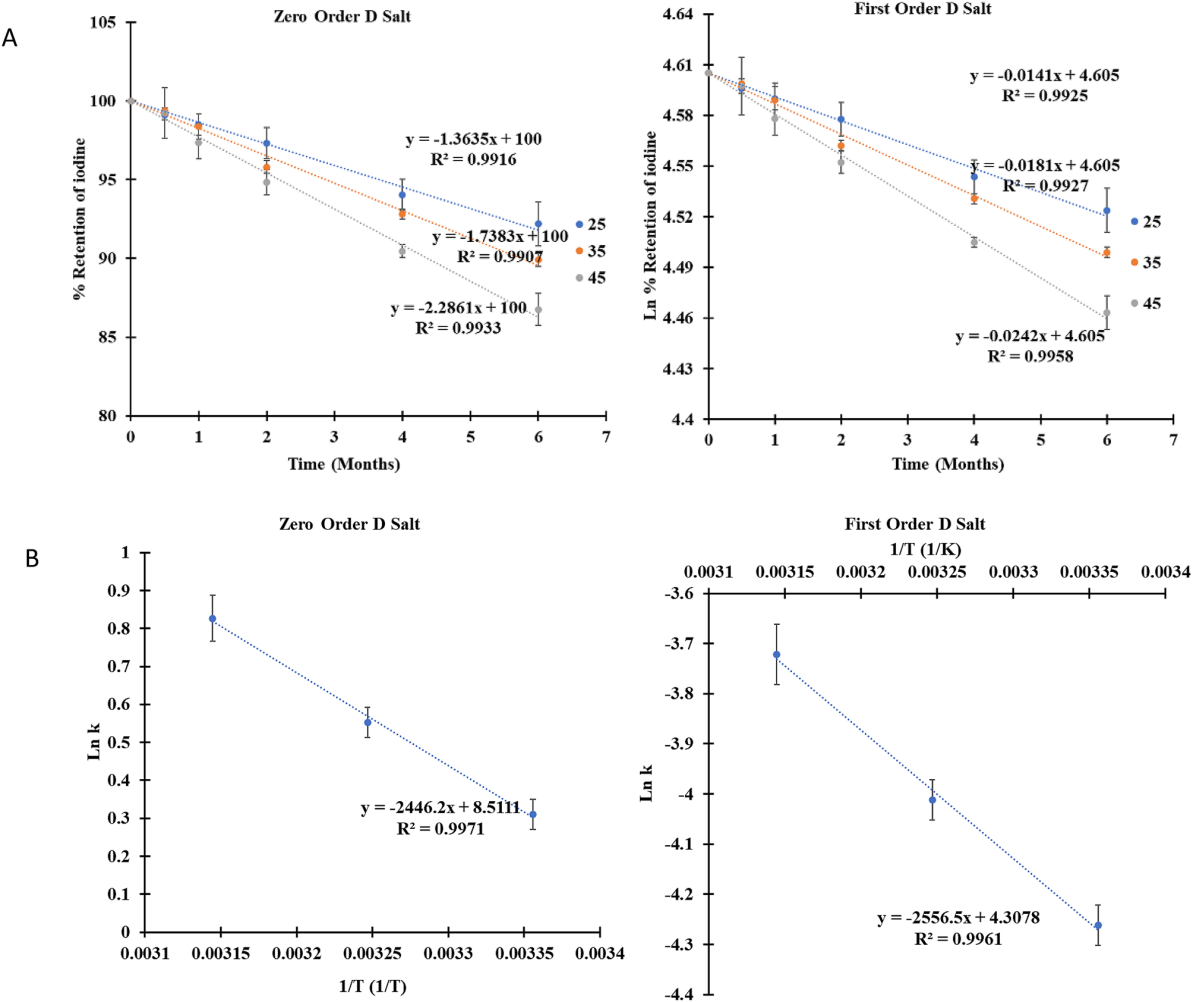
(**A**) Sample of the zero- and first-order degradation kinetics of iodine in a fortified salt; (**B**) Sample of the Arrhenius plot for the zero- and first-order degradation kinetics of iodine in a fortified salt.

Kinetic tools, such as degradation constant and Gibb free energy, are vital to predicting micronutrients' stability. The data obtained from the 6-month stability study were used to calculate the kinetic parameters for micronutrient stability in salt (Fig. 3A). The linear regression (R^2^) of the different rate laws of degradation of the micronutrients was used to predict the order of degradation.

The correlation coefficient (R^2^) of the first-order rate was slightly higher than that of the zero-order (Fig. 3A) but very much higher than that of the second-order rate. The slight difference between zero and first-order rate may be due to the two likely mechanisms of degradation of the micronutrient in the salt- diffusion through the coat of the premix and chemical interaction among the micronutrients. While the diffusion is a zero-order rate, the chemical interaction is a first-order rate. The first-order rate of degradation was chosen because chemical interaction is likely prominent of the two mechanisms. Although the activation energy (obtained from Fig. 3, Arrhenius plot, and Eq. 3) for the first-order degradation was higher than that of the zero-order, they showed similar trends (Table 4).

3 $$E_{a} = - \left( {slope~\;of\;Arrhenius\;~plot~ \times R~} \right)$$

**Table 4:** Kinetic Parameters of the Degradation of Folic Acid and Iodine in DFS* and TFS.

Salt samples	Zero order				First order			
	k (g/ml. month^-^)			Activation Energy (kJ/mole)	k (10^–2^ month^-^)			Activation energy (kJ/mole)
	25 °C	35 °C	45 °C		25 °C	35 °C	45 °C	
**Folic acid**								
C	1.8 ± 0.1	2.1 ± 0.1	2.6 ± 0.1	14.1 ± 0.7	2.0 ± 0.1	2.3 ± 0.1	3.2 ± 0.1	14.1 ± 0.7
D	2.7 ± 0.2	3.1 ± 0.1	3.7 ± 0.2	12.8 ± 0.6	3.2 ± 0.2	3.9 ± 0.1	4.6 ± 0.2	15.7 ± 0.7
CA	2.0 ± 0.1	3.0 ± 0.2	3.7 ± 0.1	27.6 ± 1.1	1.9 ± 0.1	3.2 ± 0.2	4.1 ± 0.1	30.1 ± 1.2
DA	2.3 ± 0.1	2.8 ± 0.2	3.4 ± 0.2	16.2 ± 0.9	2.8 ± 0.1	3.6 ± 0.3	4.6 ± 0.2	19.7 ± 1.1
**Iodine**								
C	0.8 ± 0	1.2 ± 0	1.6 ± 0	28.5 ± 0.8	0.8 ± 0	1.2 ± 0	1.9 ± 0.1	33.5 ± 0.8
D	1.4 ± 0	1.7 ± 0	2.3 ± 0.1	20.3 ± 0.5	1.4 ± 0	1.8 ± 0	2.4 ± 0.1	21.3 ± 0.5
CA	1.0 ± 0	1.5 ± 0.1	2.0 ± 0.1	26.4 ± 1.0	1.1 ± 0	1.8 ± 0.1	2.2 ± 0.1	26.3 ± 1.0
DA	1.0 ± 0	1.2 ± 0	1.5 ± 0	15.4 ± 0.4	1.1 ± 0	1.3 ± 0	1.9 ± 0.1	16.4 ± 0.4

The C and D salt (DFS*) are fortified by spraying folic acid and iodine solution on salt. They differ in folic acid concentration; the C salt had 12.5 ppm of folic acid + 50 ppm iodine, while the D salt had 25 ppm folic acid + 50 ppm iodine. The CA and DA salt samples had iron at 1000 ppm and their corresponding concentration of folic acid and iodine concentrations.

where E_a_ = activation energy (J/mole), R = gas constant (8.314 J/K⋅mole).

The activation energies of the C salts were higher than those of the D salts. The iron premix's presence increased the activation energies for folic acid in the salt but reduced iodine's activation energies in the salt (C vs. CA and D vs. DA, Table 1). These observations imply that folic acid and iodine were more stable in the C salts than in D salts and that iron seems to improve folic acid stability while decreasing the stability of iodine in the salts. As stated earlier, the reducing power of the ferrous fumarate may have played a role in this. While it reduces the iodate to iodine, which is then lost by sublimation, the iron may have reduced folic acid's oxidative degradation. These deductions are consistent with the trend of folic acid and iodine stability in TFS samples.

The kinetic parameters of the degradation of micronutrients in the TFS were derived based on a 6-month was validated with the stability of micronutrients in the salt in a 12-month study (Table 1). Equation 4 was derived given that the degradation of iodine and folic acid in the TFS primarily obeyed the first-order rate law. Value t_(R, T)_ being approximately equal to 12 months, obtained when R_(T)_ from twelve-month stability study and corresponding k_(T)_ from six-month stability was put into Eq. (4), validates TFS's degradation constants and Eq. (4). Using Eq. (4), the time it will take to lose 25% of the iodine and folic acid (R_(T)_ = 75%) in TFS samples was calculated. From the calculation, it will take 15, 9, and 7 months to lose 25% folic acid in TFS with the best outcome at 25, 35, and 45 °C, respectively. For iodine, it will take 26, 16, and 13 months, respectively. In all samples, the micronutrients can be projected to retain at least 75% of the added micronutrients for more than 6 months.

4 $$t_{{\left( {R,T} \right)}} = \frac{{\ln \left( {\frac{1}{{R_{{\left( T \right)}} }}} \right)}}{{k_{{\left( T \right)}} }}$$

where; t(R, T)= time (in months) required to retain micronutrient (%) in fortified salt at a given temperature, R(T)= retention of micronutrients in fortified salts (%) for a given temperature; values were obtained from the 12-month stability study, k(T)= degradation constant obtained from the 6-month stability study.

### Impact of cooking on the stability of folic acid

The impact of boiling and fermentation on the folic acid was evaluated in cooked rice and Bondi raita. There were several failed attempts for cooked rice because of the difficulty in extracting the micronutrients from cooked rice. The extraction involves sieving, and the high content of amylopectin in rice made sieving impossible. The use of sodium carbonate as a flocculant did not solve the problem. After several failed attempts, cooking rice with excess water (1:9, rice: water) helped resolve this problem. Even then, the filtration was nearly impossible. The extraction of folic acid from Bondi raita did not cause much of a problem. The fortified salt contributed significantly to the folic acid content of the two foods. Given the amount of salt added to the foods and concentration of folic acid in the unfortified and fortified cooked foods, over 70% of the folic acid due to added fortified salt was retained in the cooked rice. There was no observed sensory difference between the cooked foods with or without the fortified salt.

### Cost analysis

Since the poor and vulnerable populations, who cannot afford or do not have access to processed fortified foods, are the primary targets of this technology, there was a need to evaluate the technology's cost implication to assess if the targeted population can afford the fortified salt. The formulation of premix is the additional unit operation to the traditional process of making iodized salt. Based on the average daily consumption of 10 g salt^31,32^, the additional cost of triple fortification is about 27¢/person per year. It is assumed that this cost can be reduced further by large-scale production and be covered by government or philanthropic contributions for the needy.

## Conclusion

The chemistry and colour of folic acid guided the development of the technology for adding folic acid to the established process of double fortification of salt. Folic acid that is susceptible to oxidative degradation was more stable when coextruded with iron. Also, the coextrusion of iron and folic acid limits folic acid's impacts on the salt's sensory properties. Based on the kinetic model developed, folic acid and iodine stability in the salt are predicted to exceed six months for retention of over 75% of the micronutrients added to salt at 45 °C 60–70% RH. The additional cost for adding the three micronutrients to salt is about 27¢/person per year. More so, large-scale production will further reduce the cost. Folic acid in the salt was stable in boiling and fermentation in cooked rice and Bondi raita, respectively. The described technology is potentially a cost-effective approach for simultaneously ameliorating iron, iodine, and folic acid deficiencies.

## Supplementary Information

Supplementary Information.
