# Supplementary figures and images for: Folic acid fortification of double fortified salt
Source: Sci Rep. 2021 Jul 15;11:14561. doi: 10.1038/s41598-021-93194-9 (PMC8282793; doi:10.1038/s41598-021-93194-9)

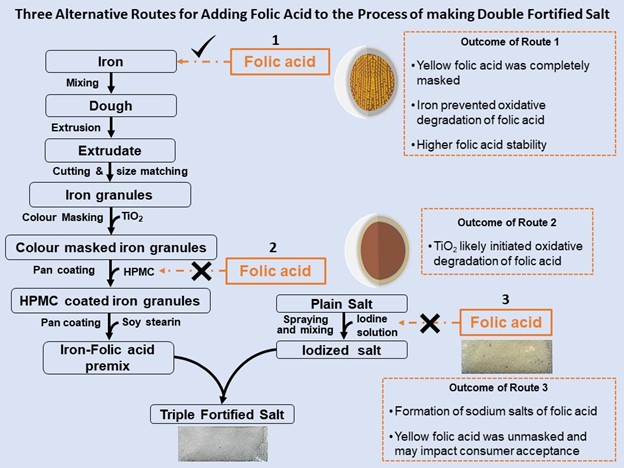

Supplement: Supplementary file 1 — Supplementary Information. [file 41598_2021_93194_MOESM1_ESM.jpg]
